# The experiences and perspectives of people with gout on urate self‐monitoring

**DOI:** 10.1111/hex.14071

**Published:** 2024-05-14

**Authors:** Toni J. F. Michael, Jian S. Chan, Stephen Hughes, Daniel F. B. Wright, Matthew J. Coleshill, Dyfrig A. Hughes, Richard O. Day, Parisa Aslani, Sophie L. Stocker

**Affiliations:** ^1^ School of Pharmacy, Faculty of Medicine and Health The University of Sydney Sydney New South Wales Australia; ^2^ St Vincent's Clinical School Campus, Faculty of Medicine The University of New South Wales Sydney New South Wales Australia; ^3^ Department of Clinical Pharmacology and Toxicology St Vincent's Hospital Sydney New South Wales Australia; ^4^ Black Dog Institute, Faculty of Medicine The University of New South Wales Sydney New South Wales Australia; ^5^ Centre for Health Economics and Medicines Evaluation, North Wales Medical School Bangor University Wales UK; ^6^ Sydney Infectious Diseases Institute, Faculty of Medicine and Health The University of Sydney Sydney New South Wales Australia; ^7^ Sydney Musculoskeletal Health, Faculty of Medicine and Health The University of Sydney Sydney New South Wales Australia

**Keywords:** adherence, gout, patient‐led, point‐of‐care, qualitative, self‐monitoring, urate

## Abstract

**Introduction:**

Gout management remains suboptimal despite safe and effective urate‐lowering therapy. Self‐monitoring of urate may improve gout management, however, the acceptability of urate self‐monitoring by people with gout is unknown. The aim of this study was to explore the experiences of urate self‐monitoring in people with gout.

**Methods:**

Semistructured interviews were conducted with people taking urate‐lowering therapy (*N* = 30) in a 12‐month trial of urate self‐monitoring in rural and urban Australia. Interviews covered the experience of monitoring and its effect on gout self‐management. Deidentified transcripts were analysed thematically.

**Results:**

Participants valued the ability to self‐monitor and gain more understanding of urate control compared with the annual monitoring ordered by their doctors. Participants indicated that self‐monitoring at home was easy, convenient and informed gout self‐management behaviours such as dietary modifications, hydration, exercise and medication routines. Many participants self‐monitored to understand urate concentration changes in response to feeling a gout flare was imminent or whether their behaviours, for example, alcohol intake, increased the risk of a gout flare. Urate concentrations were shared with doctors mainly when they were above target to seek management support, and this led to allopurinol dose increases in some cases.

**Conclusion:**

Urate self‐monitoring was viewed by people with gout as convenient and useful for independent management of gout. They believed self‐monitoring achieved better gout control with a less restricted lifestyle. Urate data was shared with doctors at the patient's discretion and helped inform clinical decisions, such as allopurinol dose changes. Further research on implementing urate self‐monitoring in routine care would enable an evaluation of its impact on medication adherence and clinical outcomes, as well as inform gout management guidelines.

**Patient or Public Contribution:**

One person with gout, who was not a participant, was involved in the study design by providing feedback and pilot testing the semistructured interview guide. In response to their feedback, subsequent modifications to the interview guide were made to improve the understandability of the questions from a patient perspective. No additional questions were suggested.

## 1 INTRODUCTION

Gout is an inflammatory arthritis characterised by periods of acute inflammation (gout flares) caused by the crystallisation of monosodium urate in the synovial fluid of joints.^1^ It is more common in men ^1^ and its global prevalence is rising.^2^ Gout is primarily a ‘silent’ condition, in that people with gout have minimal hindrance from the condition until they experience the debilitating pain of a gout flare. While these flares are transient and self‐limiting, recurrent flares can lead to long‐term disability and permanent joint damage.^3^

The risk of gout flares increases when serum urate concentrations exceed 0.36 mmol/L.^4^, ^5^ Effective urate‐lowering treatment (ULT, e.g., allopurinol) is widely available to reduce urate below the target concentrations and to therefore reduce the likelihood of gout flares. However, adherence to ULT is suboptimal, with nearly half of the people with gout discontinuing ULT within the first 6 months of initiating therapy.^6^ Reasons for poor ULT adherence include how people with gout interact with doctors about their gout, their experiences with taking their gout medication and experiences and frequency of gout flares.^7^

One method to improve gout management is to equip people to measure their urate. The rationale is that people with gout can see how their medication‐taking behaviour impacts their urate with respect to the 0.36 mmol/L target, and, therefore, become more actively engaged in disease management.^7^ Currently, measuring serum urate concentrations is conducted infrequently, with guidelines recommending monitoring every 2–5 weeks until 0.36 mmol/L is achieved, then biannually.^8^ In practice, however, clinician adherence to these guidelines is poor,^9^, ^10^ further reducing how regularly urate is monitored. Further, when urate monitoring is conducted, patients are not necessarily provided with the results, as with any blood test.^11^ This limited feedback on their urate control means people with gout are less able to contextualise how their experience of taking ULT relates to their day‐to‐day behaviour, frequency of gout flares and underlying hyperuricemia severity. This hinders their potential ability to modify their behaviour in response to changes in urate concentration.

There are parallels between the situation in gout and other chronic conditions, notably diabetes and hypertension. These chronic ‘silent’ conditions have a measurable biomarker that represents a surrogate for condition control (i.e., urate for gout, blood glucose for diabetes, blood pressure for hypertension). All three of these conditions also have commercially available point‐of‐care devices that can measure these biomarkers. Further, these conditions require regular, life‐long medication to keep their symptoms under control (i.e., ULT for gout, antihyperglycemic agents for diabetes, antihypertensives for hypertension). However, while diabetes and hypertension guidelines support patient self‐monitoring of their biomarker using a point‐of‐care device,^12^, ^13^ the application of patient‐led monitoring has not yet been extended to gout management guidelines.

Our group has shown that urate self‐monitoring for 12 months improves adherence to ULT, in conjunction with target urate attainment and reduced gout flare frequency.^14^ After self‐monitoring urate for 12 months (*n* = 32), participants engaged in an exit interviews (*n* = 30) to discuss their experiences of urate self‐monitoring and their perspective on how it could impact their gout management. This publication discusses the significance and daily impact of urate self‐monitoring on gout management from the participants’ perspective.

## 2 METHODS

In a 12‐month observational feasibility study,^14^ people with gout were required to self‐monitor urate at least once a month using a point‐of‐care device (capillary blood sample, HumaSens2.0^plus^ Multiparameter System; Human Diagnostics). Participants were told that maintaining urate concentration within target (≤0.36 mmol/L) reduces the risk of a gout flare, and received a graph of their urate concentrations monthly. Meanwhile, their adherence to allopurinol was monitored electronically (participants were aware of adherence monitoring but were blinded to data, measured using MEMS®; Aardex). At the end of the observational period, participants were interviewed about their experience of urate self‐monitoring.

The aim of this current research was to understand the perspective of people with gout who had first‐hand experience of urate self‐monitoring and their views on how urate self‐monitoring impacts gout management.

### 2.1 Study design and participants

The main study was conducted in Australia from June 2021 to April 2023. Participants were recruited through the study investigators' networks and social media advertisements. Recruitment was stratified by rurality^15^ at 1:1, metropolitan to rural. Eligible participants included people with gout who were prescribed allopurinol at study enrolment, were at least 18 years old and were proficient in the English language. People who were assisted in their medication taking, such as requiring carers or adherence aids, were ineligible to participate. Written informed consent for participation was obtained.

### 2.2 Data collection

A semistructured interview guide (Supporting Information S1: Material 1) was developed. The guide was content validated by senior researchers experienced in gout management and/or adherence (S. L. S., M. J. C., R. O. D.), and pilot tested with one person with gout. Key topics included: understanding of urate and ULT; past experiences of taking ULT; past experiences of managing their gout; experience and opinions of urate self‐monitoring; interpretation and impact of having access to urate readings; opinions on the practical aspects of urate self‐monitoring (e.g., ease of device use); opinions about the implementation of urate self‐monitoring in standard gout management.

Demographic and clinical data (e.g., age, sex, residential location, dose of allopurinol, duration of gout) were collected at study enrolment. All interviews were conducted by T. J. F. M. between 15 June 2022 and 4 April 2023, which reflected the end date of the participants' 12 months of urate self‐monitoring. Interviews were conducted through telephone or video conferencing, were transcribed verbatim and deidentified. A second investigator, S. L. S., analysed a subset of transcripts to ensure interviewing technique was appropriate, providing feedback to the interviewer accordingly.

### 2.3 Data analysis

Interview transcripts were thematically analysed inductively.^16^ Open coding was conducted iteratively by T. J. F. M., who was supported by S. H. Themes were independently identified by J. S. C., ensuring the accuracy and validity of the themes identified by T. J. F. M. through comparative analysis. Two senior investigators, S. L. S. and S. H., assisted and facilitated discussion during theme development. All authors were involved in theme refinement and subsequent manuscript development.

## 3 RESULTS

Out of the 32 participants enroled in the main study, 30 completed an exit interview, 1 withdrew (unrelated to point‐of‐care testing) and 1 was lost to follow‐up. Participant characteristics of those who completed an exit interview are described in Table 1. Interviews lasted 37 min (median, range 18–77 min). Participants were diagnosed with gout 18 years ago (median, range 6–46), and had been prescribed allopurinol for 10 years (median, range 1–41).

**Table 1:** Participant characteristics.

Characteristic	Participants (*N* = 30)
Median age at study enrolment (range), years	60 (34–86)
Male gender, *n* (%)	28 (93%)
Living in an urban^a^ area, *n* (%)	16 (53%)
Years since gout diagnosis	18 (6–46)^b^
Years since first allopurinol prescription	10 (1–41)^b^
As well as their general practitioner, had also seen a rheumatologist and/or specialist for gout,^c^ *n* (%)	10 (33%)^d^
Had urate monitoring^e^ every ≤6 months, *n* (%)	16 (53%)^d^
Allopurinol dose (mg/daily), mode (range)	300 (100–600)
Had allopurinol dose increased in the last 12 months, *n* (%)	6 (20%)
Urate concentration (mmol/L)	0.33 (0.20–0.52)
Experienced at least one gout flare in the last 12 months, *n* (%)	13 (43%)

*Note*: Data reported as median (range), unless stated otherwise.

^a^ Australian statistical geography standard remoteness area category 0.

^b^ Out of 28 participants.

^c^ In Australia, gout is typically managed through a general practitioner. In addition to general practitioner care, some people with gout may be overseen by a rheumatologist on referral by their general practitioner.

^d^ Out of 29 participants.

^e^ How often they attended a pathology clinic to have a blood test specifically for urate before participating in the urate self‐monitoring study.

Three core themes were inductively identified: urate self‐monitoring changed participants' understandings about gout; urate self‐monitoring informed gout‐related behaviours; and the perceived value of urate self‐monitoring varies. Illustrative quotes are provided in‐text, with themes summarised in Table 2.

**Table 2:** Summary of themes.

Themes	Subthemes
Urate self‐monitoring changed people's understanding about gout	Urate self‐monitoring was used to understand how behaviour impacted urate
Urate self‐monitoring informed gout‐related behaviours	Urate self‐monitoring was used to motivate people to achieve target urate and avoid pain gout flare pain by changing their behaviour Urate self‐monitoring provided evidence that adhering to allopurinol affected urate control
Ongoing value of, and preference for, urate self‐monitoring varied	Urate self‐monitoring at home was preferred Urate self‐monitoring may be particularly informative for people with uncontrolled urate There were different opinions on how frequently to self‐monitor urate People want to discuss their self‐monitored urate data with their doctor to inform allopurinol dose changes

### 3.1 Urate self‐monitoring changed participants' understanding about gout

All participants reported improved understanding of gout as a result of urate self‐monitoring. A common understanding was that there was an urate concentration to target to avoid gout flares, which coincided with learning how their behaviour influenced urate.

#### 3.1.1 Participants self‐monitored to better understand their urate concentrations

Self‐monitoring urate enabled participants to understand how different behaviours impacted urate concentration. For example, Participant 2 reported that they could monitor how their urate concentration changed in response to taking allopurinol.

If I've missed some doses, [urate self‐monitoring] particularly shows me how quickly it can, by not taking it, how quickly […] the levels can elevate. And then likewise, when I started [allopurinol], also really helped me highlight how quickly it does sort of help reduce the urate levels in the body. [Participant 2]

Self‐monitoring urate reassured participants that allopurinol was working as intended by keeping their urate low. It provided visibility to an otherwise silent condition, as articulated by Participant 11.

It's nice to see that the system is working, that the drug medication is working, [urate self‐monitoring] just confirms it on a regular basis rather than once every three months or six months, depending on when I do a blood test. It's giving you a confidence in that, ‘Yes you're doing the right thing, and taking medication’. You see, because with gout you don't always feel it, sometimes you don't feel it straightaway, the delay factor of sometimes two, three days a week, sometimes immediate. So, you're saying now, ‘shit, what did I have that gave me the gout’, and you try and think what you had the last week or two, but if you had the machine […] you know what the contraband is and what the results are straightaway. [Participant 11]

Many participants spoke of noticing how their urate concentration changed according to their diet, including consumption of beverages. For some, these experiences changed their understanding of dietary triggers of gout flares.

The more water I suppose I drink the usually the lower [the urate] goes. […] the days when I drank more water than cordial were the better days. […] I thought it was more with food and alcohol sort of thing. [Participant 4]

The ability to self‐evaluate the impact various behaviours had on their gout symptoms supported self‐assessment, as articulated by Participant 25.

If [urate is] starting to build up, you question what you've done since the last reading, what foods you've had, how much beer you've had, or what you have done or what you haven't done. […] Assess the lifestyle. [Participant 25]

Indeed, participants reported self‐monitoring urate to test triggers of gout symptoms. For example, Participant 6 spoke of how they tested their urate before and after eating asparagus, which they had previously understood as a dietary trigger.

I did do a couple of tests after eating asparagus, which is one that normally can sort me out, but there wasn't any real change [in urate]. [Participant 6]

Participants self‐monitored urate to understand their urate levels at the time of a gout flare.

The only thing that annoyed me was when I had the flare up and [the urate] didn't read any different. Like, I was expecting it to would go through the roof and it didn't. [Participant 1]

### 3.2 Urate self‐monitoring informed gout‐related behaviours

Many participants used their self‐monitored urate readings to change their behaviour for the purpose of maintaining, or attaining, a target urate concentration. They explained that they would change behaviour in response to high urate concentration due to fear of the pain from a higher gout flare risk. These behavioural changes included how they took their ULT and dietary choices.

#### 3.2.1 Urate self‐monitoring motivated participants to achieve target urate and avoid gout flare pain by changing their behaviour

All participants expressed a strong desire to avoid the pain associated with gout flares. As such, the ability to monitor their urate trend over time was important to participants as they believed rising urate cause gout flares. Self‐monitoring gave participants the chance to ‘correct’ behaviours that may be elevating their urate to try to avoid a gout flare, as Participant 23 described.

If I was to see it and my levels are going up, I'd be going ‘hang on, I'm going to be in for a lot of pain real soon if I don't correct what I'm doing now. What am I doing now that is different to last month?’ [Participant 23]

In addition, participants intentionally used urate self‐monitoring when feeling like a gout flare may be near, to again inform behaviour changes to avoid pain.

There was a few times where I felt like I was going to have a flare, and so I would test myself and if it [urate] was higher than what it normally should have been, then I just drank a heap of water and really watch my diet for the next couple of days, just to not have any trigger foods. [Participant 8]

The ability to self‐monitor urate acted as a motivator to inform behaviours that participants thought would improve their chances of attaining, and then maintaining, target urate concentrations. For example, Participant 8 explained this motivation to stay within target.

I felt like I was getting good [urate] results from the get‐go and I wanted to continue with that. So there was a little bit of self‐pushing, and if I went to get something bad to eat or that sort of thing that was always in the back of my mind that ‘well, in a week's time, you're going to have to test your urate level and you know, it's not going to be good if you do that sort of thing’. […] Motivation to stay on track, knowing that if I saw the higher numbers that I would be disappointed. So yeah, it was more of a motivational tool, as well as a management tool. [Participant 8]

Being able to self‐monitor urate provided participants with regular opportunities to assess their behaviours on achieving target urate concentration.

If you monitor something once a month, you've one opportunity to fix it. If you monitor twice a month, you've two opportunities to fix it. If you monitor it every day, you have 30 opportunities [in a month] to fix the problem. So, if you get a result and it's not what you expected, then you modify behaviour and you test again to see if your behaviour modification has had the desired result on your blood sugar level or your gout level or whatever it is. [Participant 11]

Some participants reflected on the more frequent feedback of self‐monitoring urate and their previous experiences with standard care, which generally included annual urate monitoring. Participant 29 articulated how annual urate monitoring did not provide sufficient understanding of urate variability throughout the year.

Every time we go to the doctor, it was once a year that he did [my urate], and he said, ‘Oh yes it's the same as last time, same as last time’. Well, it changed in that 12 months dramatically. […] Once a year, it's an indication that's basically under control, but it really doesn't tell you that during the year you could have had a lot higher or a lot lower. So, it's a nice thing to know that it's constant every 12 months, but [urate self‐monitoring] shows you how much it can vary. [Participant 29]

#### 3.2.2 Urate self‐monitoring provided evidence that adhering to allopurinol affected urate control

From reviewing their self‐monitored urate, participants were confident that taking their allopurinol regularly would control their gout, as recounted by Participant 10.

I've got confidence that what I'm doing at the moment's alright, and that the pill is working, so just keep doing what I'm doing. [Participant 10]

The understanding that allopurinol works to keep their urate low provided participants with the confidence to relax some of the dietary restrictions that they had in place out of fear of gout symptoms, as described by Participant 9.

I had a few beers every day [on holiday…]. I came back later that day and then had a test and […] it was only 01 more than the test I'd actually sent you three days before that. So it was just more about seeing how that worked and so I figured after all that, there wasn't really much that changed the levels at all, whenever I stopped eating meat or drinking. Which I guess meant that the allopurinol was working. [Participant 9]

Regular urate self‐monitoring also encouraged participants to continue to take their allopurinol to ensure they remain symptom free.

[Having the device] motivates you to check it regularly, and to take the medicine regularly. There's no point checking it regularly if you're not going to take your medicine regularly. […] You want to keep [the gout] away. [Participant 30]

Self‐monitored urate provided regular feedback to participants who reported previous nonadherence.

[Urate self‐monitoring] certainly has changed my attitude to it because beforehand, I could see no change in the urate level, no matter what I was doing. And no matter what [medication] I was taking, so that was rather discouraging. So, then I would stop being as compliant with the medication. Whereas now, I know that there's that magic [urate] figure, and that I've achieved it, and I can get to that, and so now I just have to be patient enough to keep going with it and keep it down at that level. [Participant 7]

### 3.3 Ongoing value of, and preferences for, urate self‐monitoring varied

All participants spoke of the value of urate self‐monitoring for themselves and for other people with gout. For example, some participants reported wanting to continue self‐monitoring urate to ensure it stayed within target. Other participants stated that after self‐monitoring urate and consistently recording within target, they no longer felt the need to self‐monitor. Regardless, these participants still recommended urate self‐monitoring for people with uncontrolled urate.

#### 3.3.1 Participants preferred to self‐monitor urate at home

When asked the ideal location of urate self‐monitoring, 26 (87%) participants responded in favour of self‐monitoring at home. Self‐monitoring at home was seen as most convenient.

I want my own device […] just for convenience, safety, and regularity of use. You've got to make it easy for people to use, otherwise they won't bother. [Participant 28]

This convenience was seen to facilitate more regular self‐monitoring, with access through healthcare settings, such as pharmacies, being seen as a barrier to frequent self‐monitoring. This was viewed as particularly important during gout flares when travel may be prohibitive, as described by Participant 6.

I have enough trouble get to the pharmacy 60 metres down the road. If I thought, ‘I should do a test’, it wouldn't happen. It wouldn't happen. I'd be sitting there, potentially at nine o'clock at night and have a bit of a sore toe […] I'd sooner having it myself, convenience would be the thing. I would, to be honest, if you said to me, ‘[Name], you can go down and get a test at the chemist’, I'd never do it. [Participant 6]

The only exception to the preference of urate self‐monitoring at home was if the cost of the point‐of‐care device was beyond the means of the participant. In such circumstances, having access to urate self‐monitoring in accessible healthcare settings such as a pharmacy was viewed as appropriate.

I find [the device] very simple to operate so unless it was very expensive, in which case maybe you just go to a pharmacy and do it, but otherwise, for convenience, home. [Participant 10]

#### 3.3.2 Participants identified people who may find urate self‐monitoring particularly informative

Most participants viewed the ability to self‐monitor urate as beneficial to ensure their gout is managed optimally. Participants with controlled urate also considered self‐monitoring as a useful tool to help other people who experience regular gout flares begin to assess their gout.

I think for someone with gout who suffers a lot of gout, then yes, [self‐monitoring] would be very beneficial because they'd be able to monitor, ‘Yesterday, what did I have yesterday?’ or ‘What do we have last week that made my urate levels skyrocket?’ [Participant 23]

In addition, participants thought the ability to self‐monitor would be helpful to inform people with gout during initiation and up‐titration of allopurinol, as described by Participant 9.

I would have liked to have done this whole thing you know, even 10 years ago when the medication was less, or as it progressed through the last 10 years of upping the medication that, if I could have done this all the time, then I would have known a lot more than I do now. [Participant 9]

#### 3.3.3 Participants had different views on how frequently to self‐monitor urate

Participants had different views on how frequently people with gout should self‐monitor urate. Some participants suggested regular monitoring was required to provide adequate feedback on their gout management to inform behaviour modifications if required, as described by Participant 17.

I'd say [self‐monitoring] once a week would have to be the absolute bare minimum, if you wanted to manage it. Otherwise, you wouldn't be able to catch yourself doing the wrong thing. [Participant 17]

Other participants thought that regular urate self‐monitoring was no longer necessary once target urate concentration was achieved, yet could be reinstated with gout symptom changes.

I reckon if I felt comfortable, I wouldn't bother [self‐monitoring]. I mean, I'd take a reading ever now and then, but if things are going like fine, I don't think I need to take too many readings. Then if things start to drift, I'd probably, as I said before, just change something and see what happens. [Participant 27]

#### 3.3.4 Discussing their urate concentrations with their doctor

A common view amongst participants was that a high urate concentration would trigger them to see their doctor about their gout management and discuss whether their dose of allopurinol was sufficient to achieve target urate concentration.

If you're monitoring it and your [urate] levels are still high, then you need to have a conversation with your GP and to find out why. And maybe they need to up the dose. [Participant 23]

Participants were comfortable discussing their self‐monitored urate concentrations with their doctor because they were perceived as the healthcare professional who had the most holistic understanding of the person's health beyond their gout. This view was reinforced by the experiences of participants who, during the main study, informed their doctor that their urate concentration was high, and consequently their doctor changed their allopurinol dose, as described by Participant 15.

When I was taking 200 mgs [of allopurinol], I spoke to the doctor and I showed him I've been keeping a record of the urate levels, and I know you told me what I've got to aim to keep it below, 3.6. […] So, spoke to him, and he just said, ‘Yeah, OK, well, we'll just increase it’. He's pretty good, my doctor. [Participant 15]

Participants said they would not make changes to their allopurinol dose without consultation with their doctor, as highlighted by Participant 24.

If I was able to do this [urate self‐monitor] for another 12 months […] and I was getting a really clear indication every week of how things were, and then that was something I'd go to my GP with and say, ‘so I've been testing myself every week. How would my body would handle dropping 50 milligrams?’, then that could be something to do, but no, I wouldn't test myself and go, ‘let's not take [allopurinol] for a month and see how I feel’. [Participant 24]

## 4 DISCUSSION

This study found that people with gout used urate self‐monitoring to inform their understanding about gout and make behavioural decisions to avoid gout flare pain. They self‐monitored urate to validate or adjust their behaviour to attain or maintain target urate. People with gout preferred to self‐monitor urate at home for convenience and only discuss urate results with their doctor, when necessary, to inform ULT dosing decisions. Our study is the first to report on the detailed experiences and perspectives of people with gout who have used urate self‐monitoring as part of gout management. This study supports further research into the implementation of urate self‐monitoring in people with gout in clinical practice, and consideration within gout management guidelines.

### 4.1 People with gout see urate self‐monitoring as a supportive tool to avoid painful gout flares

Wanting to avoid gout flares drives people with gout to improve their gout management,^17^, ^18^, ^19^ and they see urate self‐monitoring as a supportive tool. This fear of pain places value on whether a urate reading is within target or not. This behaviour is described in other self‐monitoring practices, where attaining target readings provides reassurance of condition control.^20^, ^21^ The response to an elevated urate reading is to alter behaviour, whether that be medication adherence or lifestyle choices, attempting to reduce urate before a gout flare occurs. Consistent with this, people with gout naïve to urate self‐monitoring have suggested this strategy to support their ULT adherence by motivating them to keep their urate under control.^7^ Similarly, the fear of experiencing a hyper/hypoglycaemic event,^22^, ^23^ and concerns of the long‐term consequences of poor glycemia control^24^ motivates people with diabetes to self‐monitor glucose regularly to inform their behaviours.

### 4.2 Self‐monitoring improves understanding of factors that impact urate control

Self‐monitoring urate enabled people with gout to test the impact of behaviour choices on their gout. This included identifying whether certain dietary choices (e.g., red meat, alcohol consumption) increased their urate, thereby increasing their risk of a gout flare. Similarly, people with diabetes have reported seeing their self‐recorded data as behavioural validation.^21^, ^25^ People with gout could also identify if their ULT was inadequate (e.g., recording elevated urate despite adhering to their ULT), creating an opportunity to discuss their concerns with their doctor. People with gout are keen to share their urate data and contribute to ULT regimen decisions with their doctor.^7^ This interest in shared decision‐making (i.e., patients and doctors deciding together on a treatment plan) is consistent with other chronic conditions,^26^ and the preferences of the general community, where most (62%) prefer shared decision‐making over consumerist (i.e., patient decides) and paternalistic (i.e., doctor decides) healthcare.^27^ Shared decision‐making is common practice for people with diabetes, with self‐recorded blood glucose data being used to inform (or negotiate) treatment plans between patients and their doctor.^28^ Similar processes could become routine gout management, if people with gout are able to self‐monitor urate.

### 4.3 Regular feedback on urate control supports people in managing their gout by creating opportunities for behavioural change

The practice of regular urate self‐monitoring allowed people with gout to see how much their urate fluctuated. This caused them to question the utility of only monitoring urate twice a year (standard care), as identifying behaviours impacting their urate over such long time periods was considered difficult. Consequently, the opportunity to modify behaviour to improve gout management is lost. This lack of feedback between appointments in standard care compared to self‐monitoring has also been described by people with hypertension.^29^ People with gout preferred to self‐monitor to enable them to align recent behaviour with target urate attainment. That is, each time they decided to self‐monitor urate, they created an opportunity to improve their gout management. Providing opportunities for patients to self‐assess how their urate relates to their behaviour may help overcome treatment (fear‐based) avoidance strategies, which are common in chronic conditions eliciting musculoskeletal pain.^30^

Self‐monitoring urate was viewed as beneficial to both people with controlled and uncontrolled urate. For those with controlled urate, self‐monitoring provided positive feedback to maintain their current behaviours. These people either continued to monitor regularly to ensure their urate remained controlled, or only monitored occasionally when they suspected a gout flare to be imminent. For those with uncontrolled urate, self‐monitoring gave essential feedback on the impact of behaviour modifications designed to achieve target urate. Aligned with the COM‐B Framework,^31^ by providing people with gout the ability to self‐monitor urate (capacity), they can decide when to test (opportunity), being prompted by their desire to avoid painful gout flares and/or attain target urate (motivation), thereby encouraging behavioural change. This supports people with gout to strive towards controlled urate/gout by enacting the behaviour that they perceive reduces urate and thus the chances of experiencing a painful gout flare.

Urate data obtained through self‐monitoring can only inform behaviour choices optimally if the user (or their carer) can interpret the data meaningfully. Patient knowledge of target urate is important for the successful implementation of urate self‐monitoring. Currently, patient knowledge about target urate is poor.^32^ Therefore, patient‐focused education is required alongside implementation of urate self‐monitoring to ensure the benefits are realised. This has been recognised in research for managing other chronic conditions, where the intervention includes both self‐monitoring and patient education.^33^, ^34^, ^35^, ^36^, ^37^ Establishing an awareness of urate targets facilitates goal‐setting as a motivational prompt, as people with gout alter their behaviour aiming to attain target urate. Similarly, goal‐setting is frequent in diabetes self‐management and is associated with improved blood glucose control.^38^

The ability to self‐monitor urate at home was valued highly by study participants due to convenience. This is particularly valuable to those living in rural and regional areas with limited access to healthcare services.^39^, ^40^ The use of urate self‐monitoring devices (alongside apps and support from healthcare providers) at home has been shown to be convenient, improve understanding, and communication.^41^ Further, the inability to self‐monitor at home, or if the device was too expensive, would prevent frequent monitoring. Subsidy also increases uptake of self‐monitoring practices,^42^, ^43^ with cost well‐recognised as a barrier to self‐monitoring when the devices are not subsidised.^23^, ^44^, ^45^ Therefore, ensuring urate self‐monitoring devices are affordable is essential to implementation into practice, as demonstrated with subsidy structures for self‐monitoring glucose^46^, ^47^ and blood pressure.^48^

### 4.4 Study limitations

Our participants were long‐term users of allopurinol, as such their views may not represent those of people initiating ULT (dose escalation phase). The perception of people newly starting ULT on urate self‐monitoring may differ and inform future use. Second, all participants had previous experiences with urate self‐monitoring, so their views could be more positive than someone without previous experience. Further, perhaps our participants were motivated to improve their gout management, so there may be additional benefits found by those who are less motivated which this study could not identify. However, our findings are consistent with the opinions of people with gout naïve to self‐monitoring urate.^7^ Finally, both T. J. F. M. and J. S. C. held patient‐facing roles during the main study, and the participants had monthly conversations during the main study with the interviewer. While using an interviewer who the participants were familiar with generated in depth responses, participants may have responded in a positive light due to this history.

## 5 CONCLUSIONS

People with gout used urate self‐monitoring to understand how their behaviour (especially medication adherence and diet) influenced their urate and could be modified to reduce urate concentrations and avoid gout flare‐associated pain. People with gout were thus motivated to self‐monitor regularly and stay within target urate concentration. The immediacy and convenience of receiving feedback on their urate control using a point‐of‐care device at home was highly valued. Self‐monitoring urate enables people with gout to share decision‐making with their doctor on gout management. Further research examining the cost‐effectiveness and implementation of urate self‐monitoring in clinical practice is required, as well as the role of gout education and healthcare professionals in self‐monitoring success.

## AUTHOR CONTRIBUTIONS

**Toni J. F. Michael**: Conceptualisation (supporting); data curation (lead); formal analysis (lead); investigation (lead); methodology (equal); project administration (equal); validation (equal); visualisation (lead); writing—original draft preparation (lead); writing—reviewing and editing (lead). **Jian S. Chan**: Formal analysis (equal); validation (equal); writing—review and editing (equal). **Stephen Hughes**: Formal analysis (equal); supervision (supporting); validation (supporting); writing—original draft preparation (equal); writing—review and editing (equal). **Daniel F. B. Wright**: Formal analysis (supporting); writing—review and editing (equal). **Matthew J. Coleshill**: Conceptualisation (equal); funding acquisition (lead); methodology (supporting); formal analysis (supporting); writing—review and editing (equal). **Dyfrig A. Hughes**: Formal analysis (supporting); writing—review and editing (equal). **Richard O. Day**: Conceptualisation (equal); formal analysis (supporting); funding acquisition (lead); methodology (equal); writing—review and editing (equal). **Parisa Aslani**: Conceptualisation (supporting); formal analysis (supporting); writing—review and editing (equal). **Sophie L. Stocker**: Conceptualisation (lead); formal analysis (equal); funding acquisition (lead); methodology (lead); project administration (lead); supervision (lead); validation (supporting); writing—original draft preparation (equal); writing—review and editing (equal).

## CONFLICT OF INTEREST STATEMENT

The authors declare no conflict of interest.

## ETHICS STATEMENT

This study is approved by the Human Research Ethics Committee of The University of Sydney (2021/216).

## Supporting information

Supporting information.

## Data Availability

The data that support the findings of this study are available on request from the corresponding author. The data are not publicly available due to privacy or ethical restrictions.

## References

[1] Dalbeth N , Merriman TR , Stamp LK . Gout. Lancet. 2016;388(10055):2039‐2052. 10.1016/s0140-6736(16)00346-9 27112094

[2] Safiri S , Kolahi AA , Cross M , et al. Prevalence, incidence, and years lived with disability due to gout and its attributable risk factors for 195 countries and territories 1990‐2017: a systematic analysis of the global burden of disease study 2017. Arthritis Rheumatol. 2020;72(11):1916‐1927. 10.1002/art.41404 32755051

[3] Becker MA , Schumacher HR , Benjamin KL , et al. Quality of life and disability in patients with treatment‐failure gout. J Rheumatol. 2009;36(5):1041‐1048. 10.3899/jrheum.071229 19332629

[4] Stamp LK , Frampton C , Morillon MB , et al. Association between serum urate and flares in people with gout and evidence for surrogate status: a secondary analysis of two randomised controlled trials. Lancet Rheumatol. 2022;4(1):e53‐e60. 10.1016/S2665-9913(21)00319-2 38288731

[5] Uhlig T , Karoliussen LF , Sexton J , et al. One‐ and 2‐year flare rates after treat‐to‐target and tight‐control therapy of gout: results from the NOR‐Gout study. Arthritis Res Ther. 2022;24:88. 10.1186/s13075-022-02772-3 35443675 PMC9020166

[6] Coleshill MJ , Day RO , Tam K , et al. Persistence with urate‐lowering therapy in Australia: a longitudinal analysis of allopurinol prescriptions. Br J Clin Pharmacol. 2022;88(11):4894‐4901. 10.1111/bcp.15435 35675118 PMC9795926

[7] Spragg JCJ , Michael TJF , Aslani P , et al. Optimizing adherence to allopurinol for gout: patients' perspectives. Br J Clin Pharmacol. 2023;89(7):1978‐1991. 10.1111/bcp.15657 36607199

[8] Gout. Australian Medicines Handbook Pty Ltd. Accessed May 7, 2024. https://amhonline.amh.net.au/chapters/rheumatological-drugs/drugs-gout/gout

[9] Edwards NL , Schlesinger N , Clark S , Arndt T , Lipsky PE . Management of gout in the United States: a claims‐based analysis. ACR Open Rheumatol. 2020;2(3):180‐187. 10.1002/acr2.11121 32114719 PMC7077776

[10] Wall GC , Koenigsfeld CF , Hegge KA , Bottenberg MM . Adherence to treatment guidelines in two primary care populations with gout. Rheumatol Int. 2010;30(6):749‐753. 10.1007/s00296-009-1056-7 19590874

[11] Kljakovic M . Patients and tests: a study into patient understanding of blood tests ordered by their doctor. Aust Fam Physician. 2012;41(4):241‐243. https://pubmed.ncbi.nlm.nih.gov/22574351/ 22574351

[12] Australian Government Department of Health . Australian National Diabetes Strategy 2021–2030. Australian Government Department of Health; 2021. Accessed May 7, 2024. https://www.health.gov.au/sites/default/files/documents/2021/11/australian-national-diabetes-strategy-2021-2030_0.pdf

[13] National Heart Foundation of Australia . *Guidelines for the Diagnosis and Management of Hypertension in Adults—2016*. NHF; 2016. Accessed May 7, 2024. https://www.heartfoundation.org.au/for-professionals/hypertension

[14] Michael TJF , Wright DFB , Chan JS , et al. Patient‐led urate self‐monitoring to improve clinical outcomes in people with gout: a feasibility study. ACR Open Rheumatol. 2024; Early View. 10.1002/acr2.11666 PMC11246832 38591107

[15] Australian Bureau of Statistics . *Australian Statistical Geography Standard (ASGS) Edition 3*. Australian Bureau of Statistics; 2023. Accessed May 7, 2024. https://www.abs.gov.au/statistics/standards/australian-statistical-geography-standard-asgs-edition-3/jul2021-jun2026

[16] Braun V , Clarke V . Using thematic analysis in psychology. Qual Res Psychol. 2006;3(2):77‐101. 10.1191/1478088706qp063oa

[17] Harrold LR , Mazor KM , Velten S , Ockene IS , Yood RA . Patients and providers view gout differently: a qualitative study. Chronic Illn. 2010;6(4):263‐271. 10.1177/1742395310378761 20675361 PMC3134238

[18] Singh JA . Facilitators and barriers to adherence to urate‐lowering therapy in African‐Americans with gout: a qualitative study. Arthritis Res Ther. 2014;16(2):R82. 10.1186/ar4524 24678765 PMC4060486

[19] van Onna M , Hinsenveld E , de Vries H , Boonen A . Health literacy in patients dealing with gout: a qualitative study. Clin Rheumatol. 2015;34:1599‐1603. 10.1007/s10067-014-2838-1 25501463

[20] Huniche L , Dinesen B , Nielsen C , Grann O , Toft E . Patients' use of self‐monitored readings for managing everyday life with COPD: a qualitative study. Telemed J E Health. 2013;19(5):396‐402. 10.1089/tmj.2012.0135 23531094

[21] Peel E , Douglas M , Lawton J . Self monitoring of blood glucose in type 2 diabetes: longitudinal qualitative study of patients' perspectives. BMJ. 2007;335(7618):493. 10.1136/bmj.39302.444572.DE 17761996 PMC1971180

[22] Snoek F , Malanda U , de Wit M . Self‐monitoring of blood glucose: psychological barriers and benefits. Eur Diab Nursing. 2008;5(3):112‐115. 10.1002/edn.122

[23] Ong WM , Chua SS , Ng CJ . Barriers and facilitators to self‐monitoring of blood glucose in people with type 2 diabetes using insulin: a qualitative study. Patient Prefer Adherence. 2014;8:237‐246. 10.2147/PPA.S57567 24627628 PMC3931581

[24] Baird HM , Webb TL , Martin J , Sirois FM . The relationship between a balanced time perspective and self‐monitoring of blood glucose among people with type 1 diabetes. Ann Behav Med. 2019;53(2):196‐209. 10.1093/abm/kay028 29750415

[25] Peel E , Parry O , Douglas M , Lawton J . Blood glucose self‐monitoring in non‐insulin‐treated type 2 diabetes: a qualitative study of patients' perspectives. Br J Gen Pract. 2004;54(500):183‐188. https://pubmed.ncbi.nlm.nih.gov/15006123/ 15006123 PMC1314828

[26] Pagès‐Puigdemont N , Mangues MA , Masip M , et al. Patients' perspective of medication adherence in chronic conditions: a qualitative study. Adv Ther. 2016;33:1740‐1754. 10.1007/s12325-016-0394-6 27503082 PMC5055556

[27] Murray E , Pollack L , White M , Lo B . Clinical decision‐making: patients' preferences and experiences. Patient Educ Couns. 2007;65(2):189‐196. 10.1016/j.pec.2006.07.007 16956742

[28] Montenegro RE , Dori‐Hacohen G . Morality in sugar talk: presenting blood glucose levels in routine diabetes medical visits. Soc Sci Med. 2020;253:112925. 10.1016/j.socscimed.2020.112925 32244153

[29] Hanley J , Ure J , Pagliari C , Sheikh A , McKinstry B . Experiences of patients and professionals participating in the HITS home blood pressure telemonitoring trial: a qualitative study. BMJ Open. 2013;3(5):e002671. 10.1136/bmjopen-2013-002671 PMC3657666 23793649

[30] Vlaeyen JWS , Linton SJ . Fear‐avoidance and its consequences in chronic musculoskeletal pain: a state of the art. Pain. 2000;85(3):317‐332. 10.1016/s0304-3959(99)00242-0 10781906

[31] Michie S , van Stralen MM , West R . The behaviour change wheel: a new method for characterising and designing behaviour change interventions. Implement Sci. 2011;6:42. 10.1186/1748-5908-6-42 21513547 PMC3096582

[32] Coburn BW , Bendlin KA , Sayles H , Hentzen KS , Hrdy MM , Mikuls TR . Target serum urate: do gout patients know their goal? Arthritis Care Res. 2016;68:1028‐1035. 10.1002/acr.22785 26784147

[33] Ba‐Essa EM , Mobarak EI , Alghamdi A , Al‐Daghri NM . Intensified glucose self‐monitoring with education in Saudi DM patients. Int J Clin Exp Med. 2015;8(10):19374‐19380. https://pubmed.ncbi.nlm.nih.gov/26770578/ 26770578 PMC4694478

[34] Franciosi M , Lucisano G , Pellegrini F , et al. ROSES: role of self‐monitoring of blood glucose and intensive education in patients with type 2 diabetes not receiving insulin. A pilot randomized clinical trial. Diabet Med. 2011;28(7):789‐796. 10.1111/j.1464-5491.2011.03268.x 21342243

[35] Lemozy‐Cadroy S , Crognier S , Gourdy P , et al. Intensified treatment of type 1 diabetes: prospective evaluation at one year of a therapeutic patient education programme. Diabetes Metab. 2002;28(4):(1) 287‐294. https://pubmed.ncbi.nlm.nih.gov/12442066/ 12442066

[36] Silva DDR , Bosco AA . An educational program for insulin self‐adjustment associated with structured self‐monitoring of blood glucose significantly improves glycemic control in patients with type 2 diabetes mellitus after 12 weeks: a randomized, controlled pilot study. Diabetol Metab Syndr. 2015;7:2. 10.1186/1758-5996-7-2 25904987 PMC4405992

[37] Sim KH , Hwang MS . Effect of self‐monitoring of blood glucose based diabetes self‐management education on glycemic control in type 2 diabetes. J Korean Acad Soc Nurs Educ. 2013;19(2):127‐136. 10.5977/jkasne.2013.19.2.127

[38] Fredrix M , McSharry J , Flannery C , Dinneen S , Byrne M . Goal‐setting in diabetes self‐management: a systematic review and meta‐analysis examining content and effectiveness of goal‐setting interventions. Psychol Health. 2018;33(8):955‐977. 10.1080/08870446.2018.1432760 29498547

[39] Shephard M , Shephard A , Matthews S , Andrewartha K . The benefits and challenges of point‐of‐care testing in rural and remote primary care settings in Australia. Arch Pathol Lab Med. 2020;144(11):1372‐1380. 10.5858/arpa.2020-0105-RA 33106858

[40] Wong HY , Marcu LG , Bezak E , Parange NA . Review of health economics of point‐of‐care testing worldwide and its efficacy of implementation in the primary health care setting in remote Australia. Risk Manag Healthc Policy. 2020;13:379‐386. 10.2147/RMHP.S247774 32440241 PMC7212773

[41] Riches PL , Alexander D , Hauser B , Kuske B , Krause A . Evaluation of supported self‐management in gout (GoutSMART): a randomised controlled feasibility trial. Lancet Rheumatol. 2022;4(5):e320‐e328. 10.1016/S2665-9913(22)00062-5 38294032

[42] Johnson SR , Holmes‐Walker DJ , Chee M , Earnest A , Jones TW . Universal subsidized continuous glucose monitoring funding for young people with type 1 diabetes: uptake and outcomes over 2 years, a population‐based study. Diabetes Care. 2022;45(2):391‐397. 10.2337/dc21-1666 34872983 PMC8914416

[43] Nyomba BLG , Berard L , Murphy LJ . The cost of self‐monitoring of blood glucose is an important factor limiting glycemic control in diabetic patients. Diabetes Care. 2002;25(7):1244‐1245. 10.2337/diacare.25.7.1244-a 12087028

[44] Shah SG , Barnett J , Kuljis J , Hone K , Kaczmarski R . Factors determining patients' intentions to use point‐of‐care testing medical devices for self‐monitoring: the case of international normalized ratio self‐testing. Patient Prefer Adherence. 2013;7:1‐14. 10.2147/PPA.S38328 23300344 PMC3536357

[45] Ferguson C , Hickman LD , Turkmani S , Breen P , Gargiulo G , Inglis SC . “Wearables only work on patients that wear them”: barriers and facilitators to the adoption of wearable cardiac monitoring technologies. Cardiovasc Digit Health J. 2021;2(2):137‐147. 10.1016/j.cvdhj.2021.02.001 35265900 PMC8890057

[46] National Diabetes Services Scheme . Products. Accessed May 7, 2024. https://www.ndss.com.au/products/

[47] Australian Government Department of Health . Medicare benefits schedule—item 73812. Accessed May 7, 2024. https://www.mbsonline.gov.au/internet/mbsonline/publishing.nsf/Content/Home

[48] Australian Government Department of Health . Medicare benefits schedule—item 11607. Accessed May 7, 2024. https://www.mbsonline.gov.au/internet/mbsonline/publishing.nsf/Content/Home

