# Supplementary material for: The experiences and perspectives of people with gout on urate self‐monitoring
Source: Health Expect. 2024 May 14;27(3):e14071. doi: 10.1111/hex.14071 (PMC11092534; doi:10.1111/hex.14071)
Supplement: Supplementary file 1 — Supporting information. [file HEX-27-e14071-s001.docx]

**Supplementary Material 1.** Semi-structured interview guide.

Before we begin the interview, I would like to remind you of your right to skip any question that makes you uncomfortable, and we may terminate the interview at any point if that is what you wish. There will be no consequences for either action. If you do skip a question or decide to end the interview early, you may decide whether you would like the partial recording of your interview to still be included in our analysis.

This exit interview aims to gain your perspective on your experiences of the study over the past 12-months.

- Can you describe your understanding of how urate is involved in gout?
- Can you describe your understanding of how allopurinol works?
- What did you think of being able to **test your urate levels** yourself?
- Did you find it helpful? Why/why not?
- Do you think being able to self-test your urate levels changed how you manage your gout? Why/why not?
- Did having access to your urate levels influence how you took your allopurinol, or how you approached your gout in general? Why/why not?
- What methods have you used to remember to take your allopurinol? Did this change at all during the study?
- Did you experiment using the device at all? For example, did you test your urate after eating certain foods, or did you test at different times of the day? Why?
- How did you decide when to self-test your urate levels?
- Did you follow how your urate levels changed? Why/why not? How do you think your urate levels changed over the last 12-months?
- Do you think knowing your urate levels impacted your gout medication taking behaviour? [Mention the participant’s data here]
- Do you think being able to monitor your urate levels impacted how often you had gout flares? Why/why not?
- IF RELEVANT: How did testing your urate levels compare to testing your glucose levels for managing your diabetes?
- How did you find using the urate self-testing **device**?
- What did you like/dislike about the device itself? Why?
- Did it ever malfunction, or was it difficult to use at all?
- What method did you use to record your urate levels and keeping track of them? Is there another way you would prefer to record your urate levels?
- Would it be useful if the device could send your results to your mobile, or your GP directly? Why/why not?
- Would you recommend using this device to another person with gout to monitor their urate? Why/why not?
- How much would you pay to purchase this device?
- Would you want to use it at home like you have for the past year, or would you prefer accessing the device occasionally at a pharmacy, or at your GP’s clinic? Why?
- Who would you want to discuss your urate results with? Would you ever discuss the results with your local pharmacist for advice? Why/why not?
- Would you want to continue using the device after the study finishes? Why/why not?
- Did you discuss any aspects of the study with your **GP** or another healthcare professional? Why/why not?
- How did they respond to the study?
- Did this impact your gout management plan at all?
- IF RELEVANT: What do you think made your doctor increase your dose of allopurinol?

- Has this study **changed your view** on gout management or allopurinol at all? Why/why not?
- Do you see allopurinol differently?
- Has it changed how you value or interpret your urate levels?
- Did it change your perspective on how your gout was being managed before enrolling in the study?

To finish the interview, do you have any final comments you would like to make? If you have anything to add later, please feel free to contact me.
